# Effects of Microwave Energy on Fast Compressive Strength Development of Coal Bottom Ash-Based Geopolymers

**DOI:** 10.1038/s41598-019-52160-2

**Published:** 2019-10-30

**Authors:** Sungil Hong, Hyo Kim

**Affiliations:** Department of Chemical Engineering, University of Seoul, 163 Seoulsiripdae-ro, Dongdaemun-gu, Seoul 02504 Korea

**Keywords:** Pollution remediation, Geochemistry

## Abstract

Microwave energy has been shown to be effective for geopolymer synthesis due to its fast and penetrative heating characteristics; however, the changes in the physicochemical properties of the geopolymer, resulting from the microwave irradiation, have not been fully elucidated. Therefore, this study is aimed at investigating the effect of the microwaving on the properties of coal bottom ash(CBA) geopolymers. We prepared geopolymer samples by casting a mixture of ground CBA and 14 M NaOH solution against cubic molds with a hand press machine, followed by pre-curing in a dry oven at 75 °C for 24 h and microwaving under various powers and durations. The compressive strength strongly depended on the moisture content, *i.e*., the strength increased from 21 to 65 MPa or higher as the moisture content decreased to critical values, after which the strength began to decrease. The results showed that microwave energy stimulated an additional geopolymerization by evaporating the redundant free water. This led to the strength gain, although the over-irradiation generated a high internal stress and poor structural integrity, which resulted in the strength loss. Therefore, the appropriate application of microwave energy is a promising option for synthesizing high-strength geopolymers in a cost- and time-effective manner.

## Introduction

Since the first discovery of the alkali-activated binders by V. Glukhovsky in 1959^1^, intensive efforts have been put into examining and enhancing their physicochemical properties. The term “geopolymer” was coined by J. Davidovits in the late 1970s to describe the inorganic polymeric system synthesized from the alkali activation of metakaolin^2^, and it has become the most generic term for referring to the material today^3^. It has been suggested that the framework of geopolymers consists of SiO_4_ and AlO_4_ that are tetrahedrally linked by covalently bonded oxygen atoms, while cations (*e.g*., Na^+^ and K^+^), sourced from alkaline solutions balance the negative charge of Al^3+^ in a 4-fold coordinate system^4^. Geopolymers have been shown to attain a compressive strength as high as or higher than that of Ordinary Portland cement (OPC) in an early age^5^, as well as the outstanding resistance to fire/chemical attack, and a very low permeability, which is enough to immobilize heavy metals and radioactive wastes^6,7^. In addition, geopolymers have advantages when it comes to dealing with environmental issues in comparison with OPC: not only can geopolymers be synthesized from industrial wastes or byproducts^8^, but also the emission of a greenhouse gas during the production/utilization of geopolymers is equivalent to 10–30% of the counterpart of OPC^9^. Accordingly, geopolymers have received much attention as a “green” alternative construction material. To reveal the fundamentals of geopolymer chemistry and widen its applications, many researchers have employed various analytical techniques, such as scanning electron microscopy (SEM), Fourier-transform infrared spectroscopy (FTIR), X-ray diffraction (XRD), and nuclear magnetic resonance (NMR)^4,10–15^. Especially, A. Fernández-Jiménez and his group delineated the reaction mechanism of geopolymer synthesis, so called “geopolymerization”, into several successive steps that occur while overlapping each other to some extent. They are as follows^12–14^: (1), dissolution of oligomers from the starting materials into the alkaline media; (2), polycondensation of these species to produce polymeric aluminosilicate structures; and (3), progressive incorporation of silicon into the matrix with a growth of crystal structures in a long term.

Typically, geopolymers are synthesized by exposing a mixture of aluminosilicate sources and a highly concentrated alkali hydroxide solution (frequently with an alkali silicate), to a moderate temperature condition ranging from 50 to 90 °C, for several tens of hours or up to a month^8^. Although the geopolymers can also be produced under room temperature, the thermal treatment is more favorable given that the oligomer species are more readily dissolved in the alkaline media at the elevated temperature^16,17^. The conventional curing method of geopolymer using a hot dry oven is, however, comparatively inefficient in terms of time and energy consumption. Recently, some researchers utilized an advanced, direct heating tool— a microwave oven, in place of or in combination with the dry oven^18–21^. It was shown that high-strength geopolymers can be produced more effectively because microwave energy can promote the dissolution and polycondensation of precursor species due to its penetrative and fast heating characteristics^18,19^. Admittedly, microwave energy is not a panacea for the geopolymer synthesis considering the following. First, it is hard to accurately predict the response of geopolymers to the microwave irradiation because their dielectric characteristics are highly dependent on various factors, such as the type of alkali activator and the mix proportion of the geopolymer paste^22–24^. Furthermore, the microwave heating creates an “inverse temperature profile”: the temperature is highest at the center and decreases outwards, and it can lead to “thermal runaway”^24^. In addition, if the geopolymers are exposed to an intense microwave field, the vigorous dielectric heating would cause rapid evaporation of water and the thermal shrinkage of the matrix, which can induce cracks or fractures^18,19^. Nevertheless, microwave energy still has a high potential as an effective tool for geopolymer synthesis so long as it is utilized cautiously. In the previous research^25^, we demonstrated that the coal bottom ash (CBA)-based geopolymers could attain a high compressive strength by the additional use of microwave energy after a conventional dry-oven curing process.

The focus of this study was to reveal the fundamentals of the change in the compressive strength resulting from the microwave irradiation, through examining the changes in the physicochemical characteristics of the geopolymer samples. That is, the compressive strength of the geopolymers were measured while manipulating the power and time of a household microwave oven; subsequently, the outcomes were corroborated by macroscopic observations, such as appearance inspection, temperature measurement, and moisture content evaluation as well as structural and morphological investigations by SEM, FTIR, and XRD. Simultaneously, this study proposed a practical synthesis method for high strength CBA-based geopolymers, which would extend the utilization of microwave energy on the geopolymer applications.

## Experimental

### Raw materials

**Figure 1:**
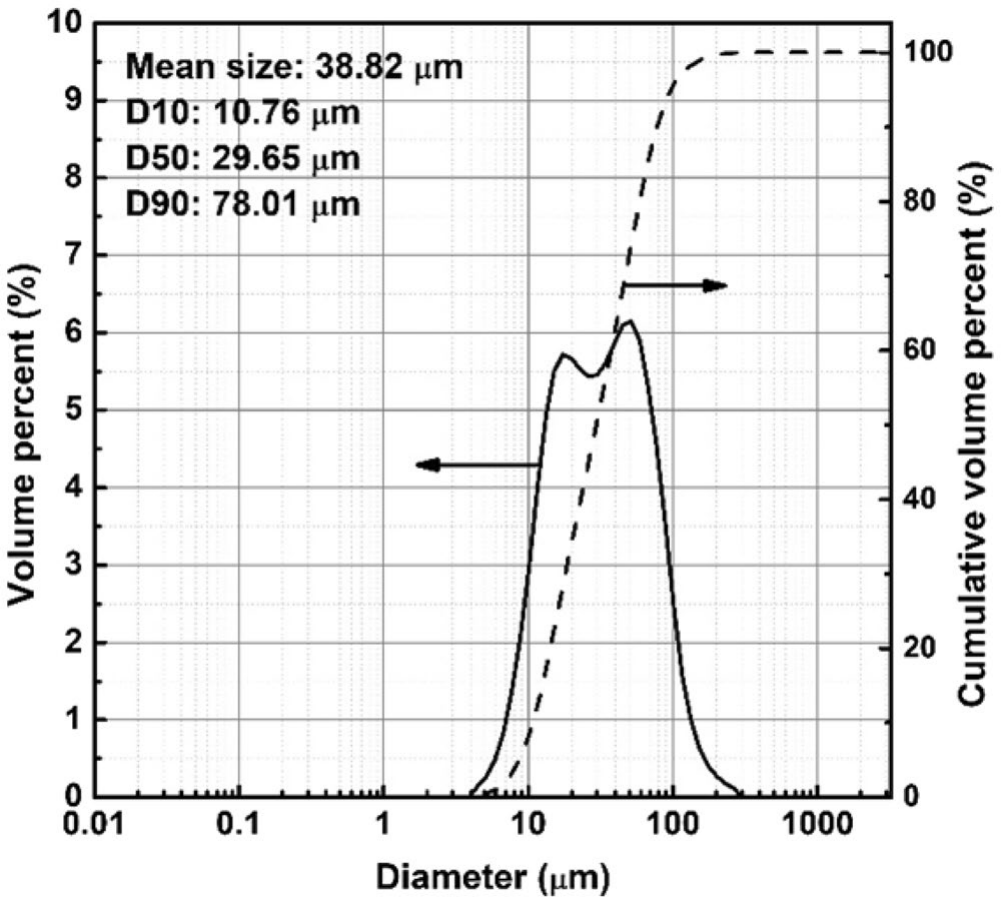
Particle size distribution of the crushed and sieved coal bottom ash (CBA).

The coal bottom ash (CBA) was provided from Yeongheung Power Plant, South Korea. It was crushed by a jaw crusher, milled by a hammer mill, and then sifted through a 200-mesh standard sieve to reduce its particle size. The particle size distribution of the sieved CBA is presented in Fig. 1 (LA-960, Horiba, Japan). The mean particle size was 38.82 μm, and 90% of the particles were below 78.01 μm. The chemical composition of CBA was analyzed in oxide formula by X-ray fluorescence (ZSK Primus II, Rigaku, Japan), and its loss on ignition (LOI) was measured in accordance with ASTM D7348, as tabulated in Table 1. The chemical composition of CBA is analogous to Class F fly ash because the sum of SiO_2_, Al_2_O_3_, and Fe_2_O_3_ is larger than 70%, as classified in ASTM C618.

**Table 1:** Chemical composition of CBA.

Component	SiO_2_	Al_2_O_3_	Fe_2_O_3_	CaO	K_2_O	TiO_2_	MgO	Na_2_O	LOI	Others
wt.%	57.6	17.0	14.5	4.14	1.27	1.17	0.85	0.74	1.76	0.97

### Geopolymer preparation

A 14 M NaOH solution was used as an alkali activator. Extra pure grade NaOH pellets (Duksan, South Korea) were added into tap water with a magnetic stirrer at the pre-determined mass ratio. After cooling the solution to the ambient temperature (20 °C), it was blended with the CBA by a Hobart mixer for 5 min at 60 rpm. The mass ratio of the alkali activator to CBA (liquid/solid or L/S ratio) was determined as 0.40 from preliminary experiments. Thereafter, the mixture of the CBA and activator was cast against 50 × 50 × 50 mm^3^ triplicate plastic molds by repeated compression with a hand-press machine. Afterward, the shaped precast samples were immediately removed from the molds, sealed in a plastic bag, and subjected to a thermal treatment at 75 °C, in a dry oven for 24 h. After this pre-curing process, the samples were unwrapped and kept standing under the laboratory conditions until they cooled to the ambient temperature. Subsequently, they were exposed to microwave energy in a household microwave oven (MW25B, LG electronics, South Korea) at 200, 400, 600, 800, or 1,000 W power. The microwave output was controlled by an inverter power supplier instead of an on-off sensor; consequently, it was able to radiate microwave energy continuously. The microwave irradiation time was set properly for each power level as suggested in Table 2. When the heating time exceeded the given ranges, the samples suffered from large cracks or even fractures, and thus became incapable of undergoing the compression test. At each condition, three geopolymer cubes were fabricated for their test values to be averaged. The samples were named corresponding to each condition as follows:

$${\circ}{\bf{W}}\,\square {\bf{M}}$$

**Table 2:** Microwave irradiation time conditions employed for each microwave power level.

Microwave power (W)	200	400	600	800	1,000
Microwave irradiation time (min)	2.5	2	1	1	1
	5	4	2	2	2
	7.5	6	3	3	3
	10	8	4	4	4
	12.5	10	5	5	5
	15	·	6	6	6
	20	·	·	·	7
	·	·	·	·	8

○ = microwave power in watts (=200, 400, 600, 800, or 1000).

□ = microwave irradiation time in minutes.

For example, 600W3M specimen is microwaved under 600 W for 3 min. Especially, the sample that was not exposed to the microwave energy will be called “Non-MW (microwave)”. In addition, for comparison of the compressive strength, we prepared two kinds of control samples: (1), after the precast samples were cured in the dry oven at 75 °C for 24 h, they were unwrapped and stored in a humid chamber at 25 °C for 6 and 20 days; the samples were labelled “Ambi-7” and “Ambi-21”, respectively; and (2), the precast samples were placed in the dry oven for 7 and 21 days without being unwrapped, and they were labelled “Oven-7” and “Oven-21”, respectively.

As mentioned above, the L/S ratio was determined as 0.40 from preliminary experiments. At the ratio, the raw mixture of the CBA and activator was in a slightly wet state, and so the precast samples were able to keep their shapes after being removed from the molds. When the alkali activator was added to CBA above this ratio, the raw mixture adopted an oozy state, which was unfavorable for the following reasons: (1), the impurities including unburnt carbon residues from the source material could gather upward and form a separate phase during the pre-curing process, which could hinder the development of the compressive strength^26^; (2), the samples become vulnerable to cracking or fracturing during the microwaving process as large amounts of water evaporate drastically^25^; and (3), the precast samples cannot be fabricated because they are unable to maintain their shapes out of the molds. Conversely, the geopolymer specimens that were prepared below the given L/S ratio showed a relatively poor compressive strength because the amount of the activator was not enough to alkali-activate the source material to a high degree. At the given L/S ratio, the precast samples were able to attain the high compressive strength without suffering from the carbon-phase separation or cracking during the hardening processes within the tested ranges. The optimal L/S ratio could be determined at the highest value as far as the precast samples were able to maintain their shapes without the molds.

### Property testing and structural characterizing methods

To study the kinetics of the weight loss of the CBA-based geopolymer involved in heating, thermal gravimetric analysis (TGA) was performed for the Non-MW sample (SDT-Q600, TA Instruments, USA). The powdered sample was heated up to 1,000 °C at a rate of 10 °C/min, and then burnt for a further 25 min. Furthermore, the derivative thermogravimetric (DTG) curve was obtained from the TGA data. To investigate the response of geopolymers to microwave heating, the surface temperature was measured by an infrared camera (E8, FLIR, USA) as soon as the microwave heating was completed. The average temperature over one side square was calculated through image processing (an average of the temperature data saved in each pixel), and the maximum and minimum surface temperatures were recorded as well. The temperature value at the regions heated beyond the measurable temperature range was estimated as 280 °C during the calculation. Subsequently, the specimens were left standing under the laboratory conditions to cool to the ambient temperature in prior to the other property tests. The moisture content, which strongly affects the physical and chemical properties of geopolymers^27^, was determined as follows. First, the moisture content of the Non-MW sample (WC_Non-MW_) was calculated by subtracting the LOI value of the Non-MW sample (=w1.3%, calculated based on the LOI estimate of CBA and the mix proportion of geopolymers) from the weight loss percent obtained from the TGA analysis (=16.1%, refer to Fig. 5). Therefore, the WC_Non-MW_ value was determined as 16.1–1.3 =14.8%. Second, the mass of water contained in each pre-cured specimen (Water_initial_) was calculated by multiplying its weight and the WC_Non-MW_ value. Third, the reduced weight of each sample during the microwave heating process was recorded as the mass of the evaporated water (Water_evap._). Thereafter, the moisture content of each sample (MC_sample_) was determined using Eq. (1)

1 $${{\rm{MC}}}_{{\rm{sample}}}( \% )=\frac{{{\rm{Water}}}_{{\rm{initial}}}-{{\rm{Water}}}_{{\rm{evap}}{\rm{.}}}}{{{\rm{W}}}_{{\rm{sample}}}}\times 100,$$

where W_sample_ is the weight of the sample. The compressive strength was measured to represent the mechanical strength of the synthesized geopolymer sample. The samples were subjected to a compression test (PL-9700H, Woojin, South Korea), in accordance with ASTM C109. To examine the micro-morphological and compositional transitions resulting from the microwave irradiation, scanning electron microscopy coupled with energy dispersive spectroscopy (SEM-EDS) was employed (Inspect F50, FEI, USA). For the SEM inspection, some pieces of the broken samples were obtained after the compression test and coated with gold once. For the comparison of the EDS quantification results, only several specific elements (Si, Al, Fe, Ca, Na, and O) were considered to give 100%. In addition, attenuated total reflectance Fourier transform infrared spectroscopic (ATR-FTIR) analysis was carried out (Frontier FT-IR Spectrometer, PerkinElmer, USA) to investigate the transitions in the chemical structure. The FTIR spectra were recorded within the range of 4000–400 cm^−1^ in transmittance with a 4 cm^−1^ resolution; however, the results were only given in 1700–400 cm^−1^ because there was no notable observation above 1700 cm^−1^ except a peak related to water. For the crystallographic analysis, X-ray Diffraction (XRD) analysis was performed (D/max-2500/PC, Rigaku, Japan). The samples of interest were finely ground and then immersed in acetone for longer than 12 h to extract structural water under the laboratory conditions. Thereafter, the samples were dried in the dry oven at 75 °C for 4 h. For the XRD reference intensity ratio (RIR) quantification, the dehydrated samples were mixed with the same quantity of the Fluorite (CaF_2_) powder (99.95% purity, Alfa Aesar, USA)^28^. The XRD patterns were recorded in a 2θ range of 5°–90°, with a step size of 0.02° per 0.6 s.

## Results and Discussion

### Appearance observation

**Figure 2:**
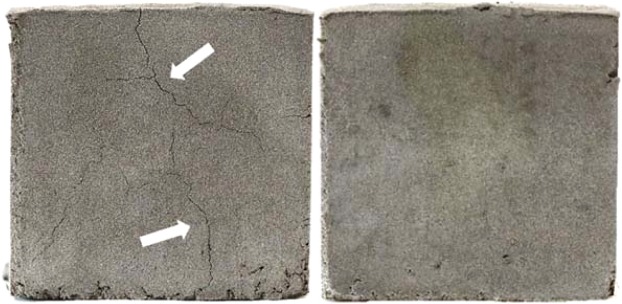
Side photographs of the 800W1M (left) and 800W5M (right) samples.

**Figure 3:**
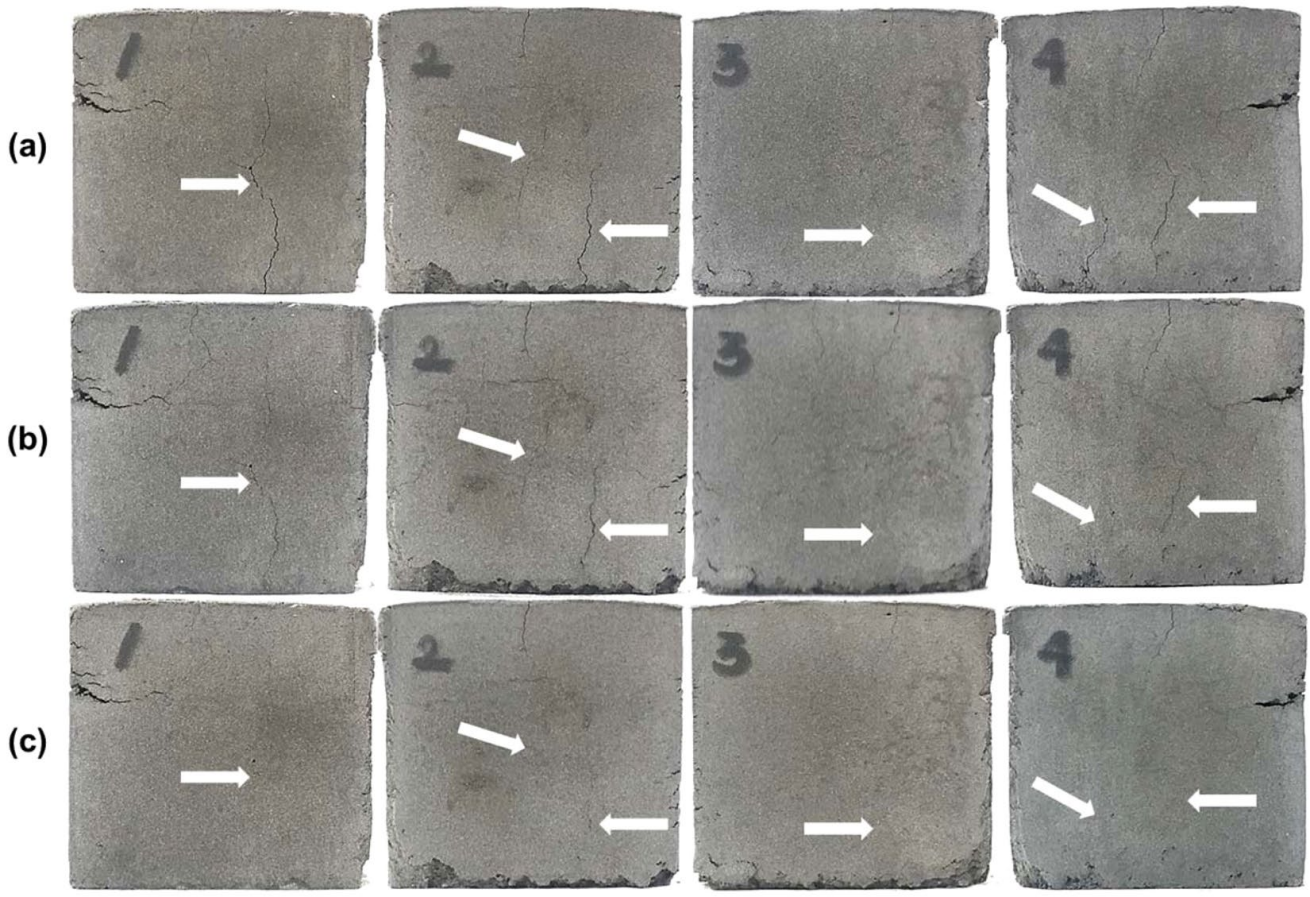
Photographs of four sides of the geopolymer specimen that was microwaved repeatedly under 200 W power. Row (**a**), after 2.5 min of the microwave irradiation; row (**b**), after the additional heating for 5 min; and row (**c**), 5 min further.

It has been reported that geopolymers are vulnerable to cracking under exposure to microwave irradiation because water vaporizes from the samples so rapidly that it brings about a very high internal stress^18,19^. At the very early stage of the microwave heating, *i.e*., the microwave irradiation time conditions noted in the second and third rows in Table 2, the hairline cracks became visible at the fronts of the samples. In contrast, the cracks were not observed for the more microwaved samples. Figure 2 shows the side images of two specimens (800W1M and 800W5M) to illustrate this comparison. This observation suggested that the longer microwave irradiation effaced the cracks, which occurred due to the rapid water evaporation at the initial heating. The supplementary experiment, where the identical specimen was repeatedly exposed to microwave energy, clarified this reasoning as given in Fig. 3. The specimen that was microwaved for 2.5 min at 200 W showed thin cracks on all the sides (Fig. 3, row (a)). Subsequently, the very same specimen was microwaved two more times, each for 5 min at the same power, and the cracks disappeared as indicated by white arrows in Fig. 3, without a perceptible volumetric expansion or shrinkage. This crack-recovery phenomenon observed in the bulk scale might have occurred because the microwave irradiation promoted additional geopolymerization reactions, which integrated the split structures. The improvement of the macroscopic integrity of the samples might have contributed to the development of the compressive strength.

**Figure 4:**
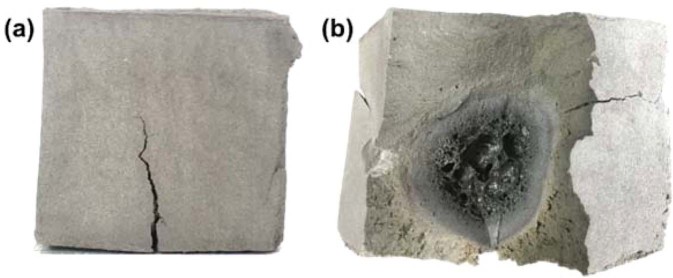
Exterior (**a**) and interior (**b**) photographs of the specimen that was fractured after being exposed to microwave energy for 9 min under 1,000 W power.

The over-irradiation with microwave, however, was detrimental to the physical properties of the geopolymers. As the samples were heated in the microwave oven over a critical point (at which the moisture content was at 4–6%, as shown in Fig. 9; it will be discussed in Section 3.4 in more detail), the compressive strength began to decrease. Even worse, the large fissures recurred on the samples when the microwave heating time exceeded the time ranges given in Table 2. Figure 4 shows the exterior and interior images of the sample that was fractured after the over-irradiation (9 min under 1,000 W power). The width of the crack was wider than that of the thin cracks at the initial heating, seen in Figs 2 and 3. The cross-sectional photograph given in Fig. 4(b) shows that severe deformation occurred at the volumetric core of the sample. A burnt and glassily bubbling morphology around the core of the sample indicated that the temperature skyrocketed as a result of the excessive microwaving; this phenomenon is called “thermal runaway”^24^. Additionally, it should be noted that some specimens that were microwaved for the longest minutes in Table 2 showed similar internal configuration, although they did not experience the external large cracks nor fractures. This inhomogeneous bulk structure was partially responsible for the deterioration of the compressive strength.

### Thermal behavior

**Figure 5:**
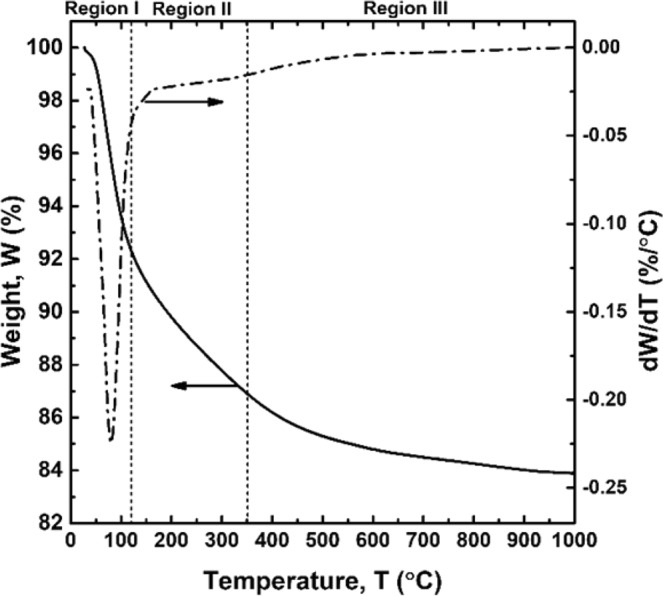
TGA (solid line) and DTG (dashed line) curves of the Non-MW sample measured in the temperature range of 25 to 1,000 °C.

TGA and DTG curves are given in Fig. 5. From the results, the thermal behavior of Non-MW geopolymer sample could be divided into three successive stages, each of which would be characterized as follows: Region I involves a rapid and substantial weight loss between room temperature and 120 °C; Region II shows a relatively slow, but significant weight loss within the range of 120 −350 °C; and Region III represents a gradual but nominal weight loss beyond 350 °C. The main factors that contributed to the weight loss in each region were different from one another. While the weight loss in Region I was primarily due to the evaporation of the physically bonded water, the loss of the chemically bonded water was mainly responsible for the weight loss in Region II, and the weight loss in the high-temperature range in Region III was attributed to dehydroxylation^15,29^. Additionally, it would be reasonable to ascribe the weight loss by LOI of the sample (=1.3%) to Region III because the decomposition of calcium compounds and combustion of residual would have occurred above 430 °C^30^. When the temperature increased to 984 °C, the residual weight reached 83.9%, and there was no additional weight change. Thus, the moisture content of the Non-MW was determined as 14.8%, by subtracting LOI of the sample from the total weight loss percentage. The key results from the TGA/DTG data are summarized in Table 3. The proportion of physically bonded water to the total water (52.0%) was not so considerable in comparison to the counterpart from previous studies (60–70%)^15,29^, because the amount of water used to synthesize the geopolymers was relatively small in this study.

**Table 3:** Summarized results of TGA/DTG.

	Weight loss (%)		Contribution to total moisture content (%)
**Region** I (25–120 °C)	Physically bonded water	7.7	52.0
**Region** II (120–350 °C)	Chemically bonded water	5.4	36.5
**Region** III (350–1,000 °C)	Hydroxyl groups	1.7	11.5
	LOI	1.3	·
Total	·	16.1	100

### Temperature increment and moisture content

**Figure 6:**
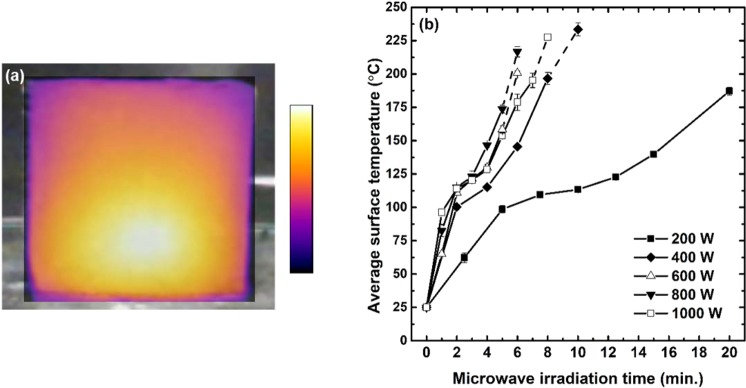
Infrared image of a microwaved sample overlapped with its side digital photograph (**a**), and average surface temperature versus microwave irradiation time profiles (**b**). Points connected with the dashed line in (**b**) indicate the cases where the temperature at the surface center exceeded the upper limit of the measurable range of the device (280 °C).

**Figure 7:**
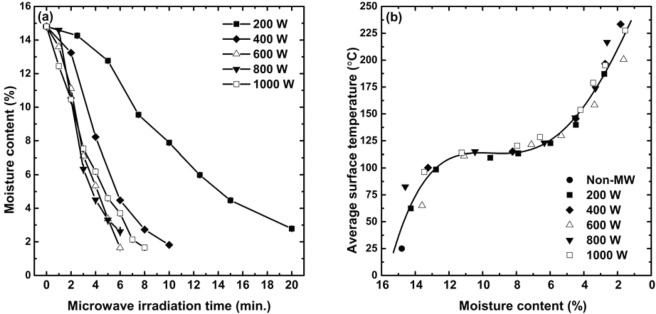
Moisture content results with respect to the microwave irradiation time (**a**), and average surface temperature results expressed as a function of the moisture content (**b**).

The entire volumes of the samples were heated rapidly in the microwave oven because the free water and ionic species in the geopolymers enabled them to absorb microwave energy and convert it to heat energy efficiently^24^. The surface temperature distribution image taken by an infrared camera is given in Fig. 6, with the average surface temperature results. Due to the vigorous dielectric heating, the average temperature increased above 100 °C only after 7.5 min under 200 W power, and the higher watts resulted in faster heating, except for the cases where the samples were microwaved at 1,000 W for longer than 2 min as shown in Fig. 6(b). Considering that the water molecules were a chief source of heat during the microwave irradiation, the overly rapid evaporation of water at the first 2 min under 1,000 W (refer to Fig. 7(a)) might have caused the slower heating thereafter. Thus, the microwave irradiation at 800 W resulted in the fastest heating rate within the tested time ranges. Figure 6(b) also shows that the average surface temperature exceeded 200 °C when the samples were microwaved for the longest minutes corresponding to each power level given in Table 2, except for the 200 W case. However, it did not mean that the entire volume of each sample was heated uniformly beyond 200 °C; in these cases, the temperature rose higher than the upper limit of the device (280 °C) at the surface center, while it was as high as 130–150 °C at the rim. In fact, the microwave heating produced a non-uniform temperature distribution where the temperature was highest at around the center and decreased outwards, as depicted in Fig. 6(a). This particular heat distribution, called “inverse temperature profile”, has been reported to occur within a few seconds when ceramics are exposed to microwave energy^31,32^. In contrast to the conventional dry-oven heating method, where heat is transferred from a surface to a core of an object by heat conduction, microwave energy heats the material throughout. However, as the samples are heated volumetrically and cooled from the outside, a temperature difference is created between the core and periphery^24^. Afterward, the dielectric loss (ε″), which represents the degree to which electromagnetic energy is converted into heat energy, becomes higher at the hotter region, and in turn, it leads to the much faster heating at the core than the surroundings^18,24^. Due to this positive feedback between the dielectric heating and property, the temperature skyrockets at the core. Given that the infrared camera is only able to measure the surface temperature, we could guess that the temperature at the volumetric core must have risen much higher than that at the surface center (max. 280 °C), and this drastic dielectric heating led to the thermal runaway as we have seen in Fig. 4(b).

The samples were reduced in weight after being microwaved due to the evaporation of water. The moisture content results calculated from Eq. (1) are given in Fig. 7(a). The higher microwave power resulted in the faster weight loss; thus, the moisture content decreased to as low as 1.6% within the tested conditions. The moisture content results, depicted with respect to the microwave irradiation time, looked like the upside-down symmetric pattern of the average surface temperature seen in Fig. 6(b), which suggested a strong correlation between the two properties. This speculation became evident as the average surface temperature results were expressed as a function of the moisture content. Figure 7(b) shows that the temperature increment was so closely related to the moisture contents, that the average surface temperature results followed the same pattern, regardless of the microwave power level. The samples were heated rapidly up to 100 °C at the initial stage of the microwave heating; however, the heating rate decreased as the temperature reached 120–130 °C, at which the moisture content fell below 6% due to the evaporation of the physically bonded water, and further heating made the average temperature soar again beyond 200 °C, which resulted in the evaporation of the chemically bonded water. This finding suggested that the physically bonded water was important for the thermal stability of the geopolymers under microwaving, in that it prevented the body temperature of the samples from jumping abruptly. Moreover, it was revealed that the microwave power level did not play a distinguishable role in determining the physical properties of the geopolymers, except that the lower power required the longer time to obtain the similar moisture content.

**Figure 8:**
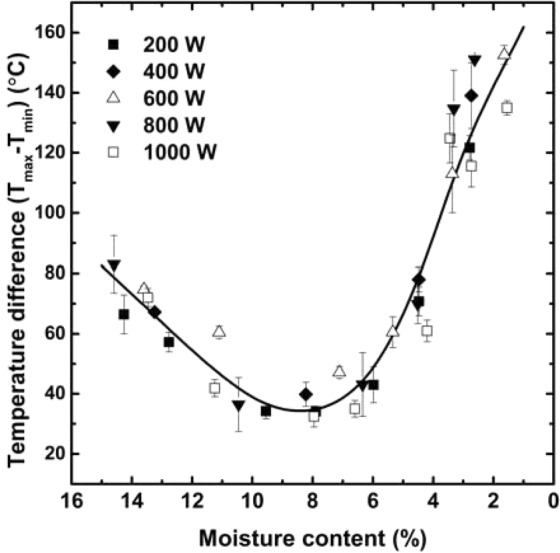
The difference between the maximum and minimum temperatures on the samples’ surface expressed as a function of the moisture content.

As described above, the geopolymer specimens came to have an inverse temperature profile due to the distinctive heating characteristics of microwave energy. Moreover, the average surface temperature and moisture content were closely related to each other, while the microwave output was not meaningful. These observations could be summarized into Fig. 8, which demonstrates the difference between the maximum temperature (T_max_, at the surface center) and minimum temperature (T_min_, at the rim) of each sample with respect to the moisture content. At the initial stage of microwaving, the temperature at the surface center was higher than the minimum temperature by 60–80 °C, as the inverse temperature profile created. However, the temperature gap decreased as the samples were further exposed to microwave energy because the temperature rose slowly at the center owing to the ongoing evaporation of the physically bound water, whereas the other part increased in temperature rapidly. After the moisture content became lower than 6%, the temperature difference began to increase again because the physically bonded water was completely evaporated at the center, while it was in progress at the outer region. Consequently, the temperature gap jumped to 110–160 °C; and then, the additional irradiation resulted in the thermal runaway. The effect of the power level of the microwave energy on the results was still imperceptible.

### Compressive strength

**Figure 9:**
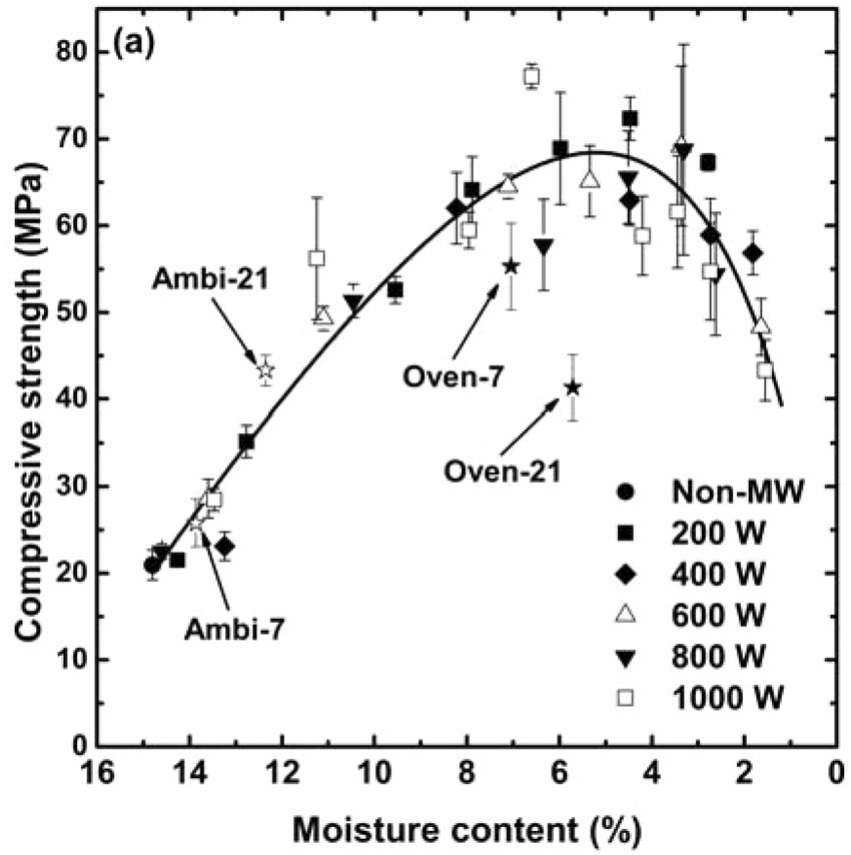
Compressive strength results of the CBA-based geopolymers with respect to the moisture content.

In the previous research^25^, it was shown that microwave energy effectively enhanced the compressive strength of pre-cured CBA-based geopolymers, by evaporating redundant free water from the matrix. Additionally, there was a critical weight loss percent value, after which the compressive strength began to decrease as the over-evaporation of water induced serious thermal stress^25^. To corroborate these observations, the compressive strength outcomes obtained in this study are expressed with respect to the moisture content in Fig. 9. The results clarified the strong correlation between the compressive strength and moisture content: as the moisture content reached the critical value at around 4–6%, the compressive strength increased by more than three times in comparison to the value of the Non-MW sample (from 21.0 to 65–70 MPa), regardless of the microwave power level (called “strength-gaining period”); however, further irradiation resulted in the loss of the strength (called “strength-losing period”). It should be noted that the critical moisture content was attained only after a short time of microwave irradiation (*i.e*., 12.5–15 min at 200 W, and 4–6 min at the higher powers; the optimum microwaving time for each power can be easily identified by comparing Figs 7(b) and 9). The compressive strength development via the microwave heating turned out to be very efficient when the results were compared to the compressive strength of the control samples as depicted in Fig. 9. As we have seen elsewhere^4,27,33,34^, the compressive strength of the hardened geopolymer samples increased continuously over a long term under the ambient temperature. Nevertheless, the compressive strength reached only 43.3 MPa even after 21 days (Abmi-21), which was much lower than the strength achieved by the microwave heating. The extended dry-oven curing under the elevated temperature (75 °C) for 7 days resulted in the higher compressive strength than that from the ambient curing, although it was also not as high as that of the microwaved samples. However, as the samples were exposed to the high temperature for the long period, the compressive strength showed deterioration rather than improvement (Oven-21, 41.3 MPa). As a result, the trend of the compressive strength vs. moisture content of the oven-cured control samples was different from that of the experimental samples. The large cracks that occurred as the long period of heat treatment broke down the geopolymeric granular structures, were responsible for the deterioration of the compressive strength^35^.

As described above, the compressive strength and moisture content results had a strong correlation, which clearly showed that water played an indispensable role in determining the physical properties of the CBA-based geopolymers. Previous researchers have also reported that the role of water is so essential that not only the macroscopic properties of geopolymers, but also their chemical/mineralogical characteristics highly depend on it^27,29,36–38^. In geopolymer chemistry, water acts primarily as a solvent of an alkaline reagent (*e.g*., sodium hydroxide, potassium hydroxide, and sodium silicate), which is required to alkali-activate the source materials^27^. Furthermore, during the dissolution stage of the source material, water participates in the reaction as a reactant, as shown in Eq. (2):

2 $$-\begin{array}{c}/\\ {\rm{T}}\\ /\end{array}-{\rm{O}}-\begin{array}{c}/\\ {\rm{T}}\\ /\end{array}-\,+{{\rm{H}}}_{2}{\rm{O}}\,\mathop{\to }\limits^{{{\rm{OH}}}^{-}}\,2\,-\begin{array}{c}/\\ {\rm{T}}\\ /\end{array}-{\rm{OH}},$$

where T = Si or Al; thus, the precursor species come to be present in hydroxylated forms, such as Si(OH)_2_, Si(OH)_4_, and Al(OH)_4_^12,27,39,40^. Subsequently, as the reaction proceeds, those dissolved species get into a polycondensation stage resulting in polymeric T-O-T chains, leading to the production of three-dimensional and somewhat disordered structures, and water re-generates as a reaction product^4,27^. Going through these stages, water became present in the geopolymer system in several different forms as briefly mentioned in Section 3.2: (1), physically bonded water (or free water); (2), chemically bonded water (or zeolitic/interstitial/structural water); and (3), hydroxyl groups^15,29,41^. Physically bonded water occupies large pores or intergranular/surface sites, and can evaporate from the matrix at a relatively low temperature range (from ambient temperature to 100 °C (or up to 150 °C, depending on studies))^15,29,36,39^. It has been reported that the compressive strength of geopolymers could be improved by getting rid of free water because it would promote an additional polycondensation reaction by eliminating the reaction product in Eq. (3)^42^.

3 $$\begin{array}{c}{\rm{n}}{({\rm{OH}})}_{3}{\rm{Si}}-{\rm{O}}-{{\rm{Al}}}^{-}{({\rm{OH}})}_{2}-{\rm{O}}-{\rm{Si}}{({\rm{OH}})}_{3}\,\\ \,\,\,\,\underrightarrow{{\rm{NaOH}}}\,{(-{\rm{SiO}}-{\rm{O}}-{{\rm{Al}}}^{-}{\rm{O}}-{\rm{O}}-{\rm{SiO}}-{\rm{O}}-)}_{{\rm{n}}}+4{{\rm{nH}}}_{2}{\rm{O}}.\end{array}$$

In this regard, the mechanism involved in the strength-gaining period would be ascribed to the elimination of the redundant free water, which would stimulate the geopolymerization reaction^25^. In contrast to the physically bound water, the chemically bound water plays a crucial role for the dimensional stability of the geopolymers, because it exists in the matrix hydrating the cations that balance the negative charge of Al in a 4-fold coordinated system^27,36,38,39^. The elimination of this kind of water, therefore, would induce a significant volumetric shrinkage^36^, leading to a high internal stress and the loss of the compressive strength^43,44^. As discussed in Section 3.3, the chemically bonded water began to escape from the geopolymers when the moisture content fell below 6%, at which point the samples also entered into the strength-losing period, as given in Fig. 9. Therefore, it would be reasonable to attribute the loss of the compressive strength to the high internal stress, which was induced by the evaporation of the chemically bound water that occurred as the geopolymer samples were overly microwaved.

It is worth comparing the data shown in Fig. 9 with the results from previous study^25^, where the CBA-based geopolymers achieved the highest compressive strength of 34.9–41.5 MPa as a result of pre-dry-oven curing at 75 °C and post-microwave heating under 700 W power. First, the highest compressive strength was attained when the precast samples were reduced in weight by 13–14%. In comparison, the compressive strength was highest when the precast samples lost 14.3–15.9% of their weights in this study. Although the optimum weight loss was somewhat higher here than in the previous study, the results agreed well with each other considering that more amount of the alkali activator was used here (L/S = 0.40; whereas, L/S = 0.38 in the earlier study). Next, the maximum compressive strength was much higher in this study (65–70 MPa) than in the previous one in spite of the similar synthesis procedure. This could be ascribed to the better quality of the CBA used herein, namely: (1), the particle size was smaller, which could increase the reaction rate^41^ (mean size of 38.82 μm, compared to 44.57 μm in the previous study); (2), the amorphous phase content was higher, so the source material participated in the reaction more actively^40,45^ (much larger amorphous hump in the XRD spectrum. Refer to Fig. 15 and the experiment section in the previous paper); (3), the amount of unburnt carbon residue, which can hinder the development of the compressive strength of geopolymers^46^, was smaller (LOI  = 1.76% here compared to 2.28% in the previous one); and (4), the molar ratio of Si to Al was higher, which helped the geopolymer products achieve the high compressive strength^47^ (Si/Al = 1.69 compared to 1.25 in the previous study). These comparisons apparently show that not only the moisture content but the quality of the source materials strongly influence the strength.

### Structural characterization

To support each mechanism of the strength-gaining and losing periods in the morphological, chemical, and mineralogical aspects, SEM-EDS, ATR-FTIR, and quantitative XRD analyses were performed.

#### Strength-gaining period

**Figure 10:**
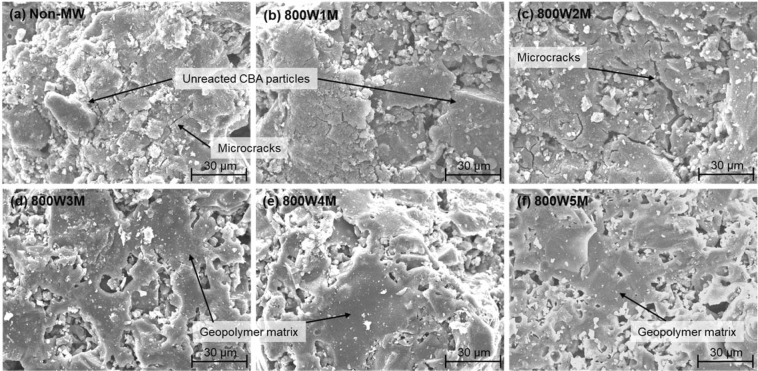
SEM images of the Non-MW sample and the samples that were microwaved under 800 W power level for 1–5 min.

It has been reported that remarkable micro-morphological transitions occur in geopolymers as the geopolymerization reaction progresses in such a way that the unreacted source material particles are attacked; thus, the reaction products interconnect the granules to improve the structural homogeneity, leading to an increase in the mechanical strength^12,14,45,47^. Figure 10 shows SEM images of the Non-MW sample and the samples that were microwaved under 800 W for up to 5 min. Many unreacted CBA particles were identified in the Non-MW and 800W1M samples (Fig. 10(a,b)), which were indicative of the relatively low degree of alkali-activation. Furthermore, some micro-cracks, which might have been due to the thermal expansion during the dry-oven curing^45^, were observed in the overall structures of them. After 2 min of microwaving (800W2M), the microstructural homogeneity was somewhat improved while micro-cracks were still observed. These morphological characteristics were partially responsible for the relatively low compressive strength of those samples. As the geopolymers were further exposed to microwave energy, however, they experienced significant changes in the microstructures in a way that well-connected geopolymer matrices were more produced to improve the homogeneity, as indicated by arrows in Fig. 10(d–f). In addition, there was no evidence of micro-cracks in those samples. The improvement in the morphology by the effective microwave heating led to the enhancement of the compressive strength^14^.

**Figure 11:**
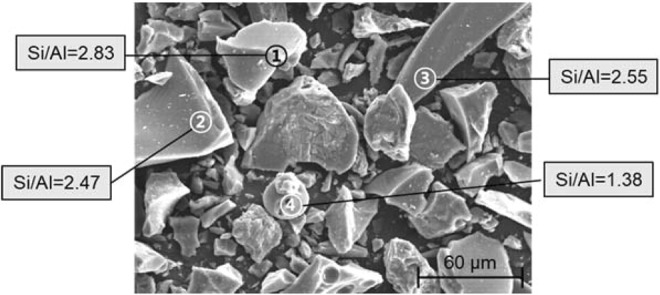
SEM image of the CBA source material and the molar ratio of Si/Al obtained from the SEM-EDS quantification, measured at four different points in the image.

For the elemental analysis of the produced geopolymeric matrices, SEM-EDS quantification was performed over the whole images seen in Fig. 10. The atomic percent results were summarized in Table 4 with the calculated molar ratio of Si/Al. A. Fernández-Jiménez and his group made an in-depth researches on the evolution of geopolymerization and the consequent Si/Al molar ratio as follows^12–14^: Al-O bonds in the source materials are more easily broken than Si-O bonds under the alkaline attack; consequently, the aluminum species get involved in the polycondensation much faster to produce the Al-rich phase products, and therefore, the Si/Al ratio cannot achieve a high value at the early stage of the reaction^14^. However, as the geopolymer matrix becomes compacted along with the progress of the reaction, further dissolution and re-polymerization of the Si-O species occur, resulting in the increment in the Si/Al ratio (Si-rich phase)^12,14^. Thus, the augmentation of the Si/Al molar ratio would represent the evolution of the reaction. As given in Table 4, the Si/Al ratios of the inspected geopolymer samples were in the range of 2.99 and 3.45. These values were higher than that of the source material (=2.88 from XRF in Table 1, or 1.38–2.83 from SEM-EDS in Fig. 11), which suggested that the reaction had progressed to the high degree after a single day of curing in the dry-oven^14^. However, there was no consistent trend in the Si/Al ratio corresponding to the microwave heating time. The absence of the trend was inevitable due to the nature of the source material. The SEM image of CBA and its EDS quantification results, which were measured at four different points, revealed that the Si/Al ratios considerably varied from one point to another (refer to Fig. 11). This high inhomogeneity in the chemical composition of CBA made the quantitative elemental analysis of the CBA-based geopolymer matrices ineffective.

**Table 4:** SEM-EDS results of the geopolymer samples given in Fig. 10 (in at.%).

Sample	Si	Al	Fe	Ca	Na	O	Si/Al
Non-MW	23.94	7.09	3.86	1.56	12.37	51.18	3.38
800W1M	22.73	6.62	4.89	1.36	13.94	50.46	3.44
800W2M	21.44	7.17	3.10	1.22	13.97	53.09	2.99
800W3M	23.81	7.41	2.89	1.28	13.04	51.58	3.21
800W4M	24.32	7.38	2.91	1.28	11.08	53.04	3.30
800W5M	25.83	7.49	2.93	1.40	11.97	50.38	3.45

**Figure 12:**
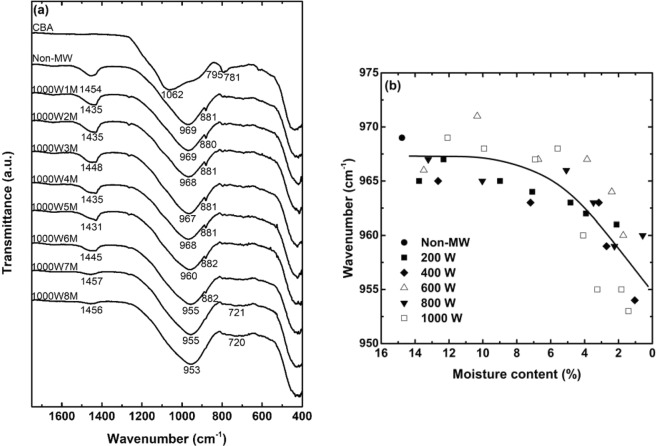
FTIR spectra of the CBA, Non-MW sample and the samples that were microwaved under 1,000 W power level for 1–8 min (**a**), and all the wavenumber values of the T-O asymmetric stretching vibration peaks with respect to the moisture content (**b**).

ATR-FTIR is a simple but powerful technique for the characterization of the aluminosilicate functional groups involved in geopolymerization chemistry. Figure 12(a) shows the FTIR spectra of CBA, Non-MW sample, and the samples that were microwaved for 1–8 min at 1,000 W. In the spectra, a broad, intense peak which represents the asymmetric stretching vibration of T-O (T = Si or Al), was detected in the wavenumber range of 800–1,200 cm^−1^ ^15^. The peak characterized at 1,062 cm^−1^ with shoulders around 1,160 and 850 cm^−1^ was observed in CBA, the pattern of which revealed the highly disordered aluminosilicate structure of the source material^48^. After the dry-oven curing process for a day, the peak decreased in the wavenumber by more than 90 cm^−1^ (from 1,062 to 969 cm^−1^), and the shoulders almost disappeared. These changes demonstrated the obvious transition in the aluminosilicate structure during the reaction, which is the reason that this peak has been called the “fingerprint” of geopolymers^11,15^. Along with the increment in the microwave heating time, it was observed that the wavenumber of the fingerprint peak gradually deceased from 969 to 953 cm^−1^. Likewise, the microwave energy shifted the wavenumber of the fingerprint peak to a lower value at all the microwave power levels, as shown in Fig. 12(b). Considering the Al-O bonds are weaker than Si-O bonds^14^, the decrease in the bond energy represents the increment in the portion of the polymeric Al-O bonds in the matrix. Therefore, this shift could be ascribable to these two mechanisms, both of which would result in the progress of the reaction and development of the compressive strength, as follows: (1), depolymerization of the silicate structure from the source material; and (2), further incorporation of aluminum into the geopolymeric backbone^11,12,49^. Given that both reactions belong to the early stage of the geopolymerization^12^, the microwave heating seemed to initiate the additional geopolymerization.

From the FTIR spectrum of CBA, the presence of quartz and cristobalite was identified as small peaks at 795, 781, and 695 cm^−1^, and a shoulder at 1160 cm^−1^ ^50^. These crystalline peaks became weak after the reaction, which indicated that the crystals were partially involved in the geopolymerization rather than remaining intact^51^. Furthermore, the peaks related to the Na_2_CO_3_ that was produced by the atmospheric carbonation of excessive sodium were observed at around 1450 and 880 cm^−1^ in the spectra of the samples^38,52^. The intensity of these peaks decreased along with the increase in the microwaving time, which suggested that microwave energy promoted further geopolymerization reaction, consuming the excessive sodium before its carbonation^25^. Finally, the nominal change was observed around 770–660 cm^−1^, which is related to the symmetric stretching vibration of T-O-Si groups^53^, after the relatively long period of microwave irradiation as shown in the spectra of the 1000W7M and 1000W8M samples. It shows that the microwave overheating induced the changes in the aluminosilicate structures.

**Figure 13:**
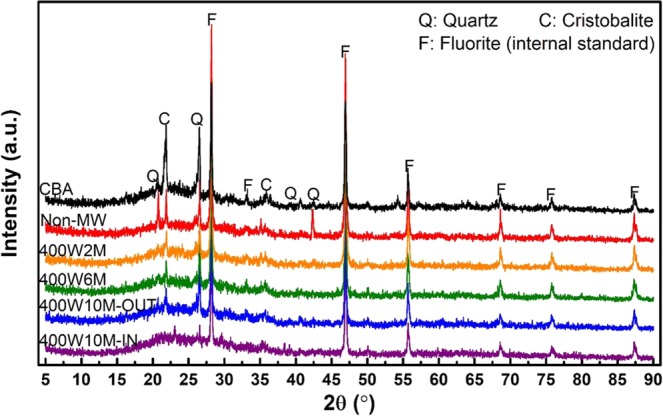
XRD spectra of CBA, Non-MW sample, and the samples that were microwaved under 400 W power for 2, 6, or 10 min (in the case of 400W10M, the analysis was performed for each region of the periphery (“OUT”) and core (“IN”)).

XRD analysis was also employed to analyze the crystallographic changes. The XRD spectra of CBA, Non-MW sample and the geopolymer samples that were microwaved at 400 W for 2, 6, and 10 min are given in Fig. 13; here, the results for 400W10M sample belong to the analysis for the strength-losing period, as described in the following section. A large, broad hump between 2θ = 15° and 35° in the spectrum of the CBA indicated the presence of a vitreous phase, which was dissolved into the alkaline media and then participated in the polycondensation^40,45^. Additionally, the crystalline peaks of quartz and cristobalite were detected in both the source material and geopolymers. The alkali activation and curing processes did not induce a change in the qualitative crystallographic results; that is, there was no formation, disappearance, nor transformation in the crystalline species. However, the RIR-XRD results revealed that the quantitative transition occurred considerably. As suggested in Table 5, the quantities of the crystal phases were reduced significantly after a single day of dry-oven curing (Non-MW), resulting in the highly amorphous matrix. This observation shows that not only the vitreous phase, but also the crystalline phases were involved in the geopolymerization^51^, as also indicated by the FTIR. While there was no change only after 2 min of the microwave irradiation, the further heating reduced the content of the amorphous phase (400W6M). This means that the recrystallization occurred as the body temperature increased to the high level^54^, which led to the development of the compressive strength^55^.

**Table 5:** Quantitative RIR-XRD results for each spectrum in Fig. 13.

Wt.%	Quartz	Cristobalite	Amorphous
CBA	26.7	24.5	48.8
Non-MW	4.2	1.9	93.9
400W2M	4.2	1.6	94.2
400W6M	9.1	1.8	89.1
400W10M-OUT	16.8	2.8	80.4
400W10M-IN	1.2	.	98.8

#### Strength-losing period

**Figure 14:**
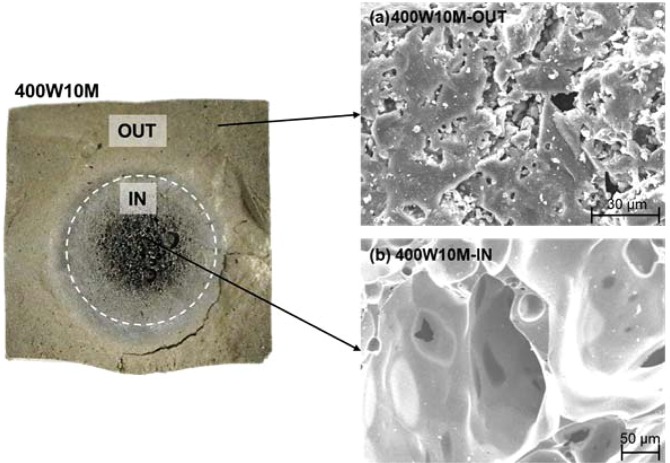
Cross-sectional image of one of the 400W10M specimens, where internal deformation occurred; and SEM images of outer (“OUT”, (**a**)) and inner (“IN”, (**b**)) regions.

**Figure 15:**
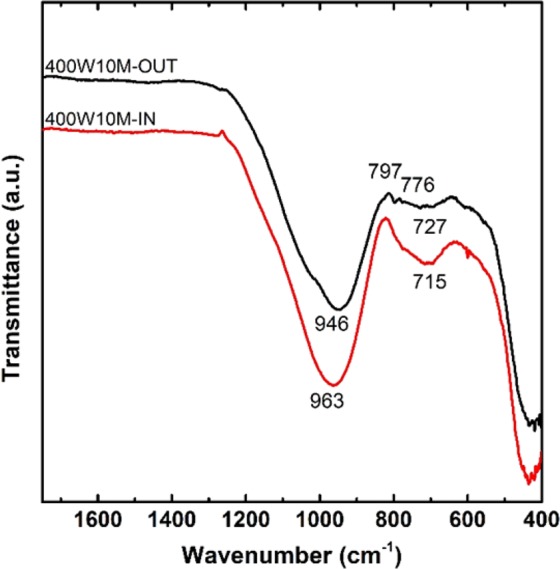
FTIR spectra of the outer (“OUT”) and inner (“IN”) regions of the 400W10M specimen that were shown in Fig. 14.

In Section 3.1, it was discussed that samples that were microwaved beyond the tested range in Table 2, suffered from the large external cracks and thermal runaway. Furthermore, several specimens belonging to the strength-losing period have experienced internal deformation as well. Figure 14 shows the cross-sectional image of one of the 400W10M specimens, where severe deformation occurred, with the SEM images of both the peripheral and core regions. The micro-morphology of the outer region (Fig. 14(a)) was quite similar to that of the 800W5M sample (Fig. 10(f)), in that the geopolymer matrix well developed to achieve the improved structural homogeneity. On the other hand, the inner region showed a very distinctive structure with a lot of large, interconnected pores and a smooth texture. In addition, there was a discrepancy in the elemental composition between these two regions as summarized in Table 6. The Si/Al molar ratio and Na content were somewhat lower at the core in comparison to each value at the peripheral region. These results implied that the aluminosilicate structure formed at the core by the microwave over-irradiation was considerably different from that of the outer region. The FTIR and XRD results also showed the difference between the chemical structures of two regions. As given in Fig. 15, the energy state of the T-O asymmetric stretching bond was higher at the core (wavenumber of 963 cm^−1^) than at the periphery (946 cm^−1^), despite the lower value of the Si/Al molar ratio. Additionally, at the core, not only was the intensity of the peak related to the symmetric stretching vibration of T-O-Si groups (around 720 cm^−1^ ^53^) much higher, but also, there was no any trace of crystalline peaks. Figure 13 shows that the XRD spectrum of the inner structure (400W10M-IN) shows a much larger hump between 2θ = 15° and 35° than that of the outer one (400W10M-OUT), which indicates the higher content of the amorphous phase at the deformed core. According to the quantitative XRD results in Table 5, the core structure was almost completely amorphous, whereas the outer region contained a considerable quantity of quartz and cristobalite. All the analytic techniques strongly showed that the microwave overheating generated the significant discrepancy between the volumetric core and periphery, and the inferior structural integrity was responsible to the deterioration of the compressive strength.

**Table 6:** SEM-EDS results measured at four different points in the outer (“OUT”) and inner (“IN”) regions of 400W10M specimen.

Sample		Si	Al	Fe	Ca	Na	O	Si/Al
400W10M-OUT	1	26.34	7.66	3.48	1.33	12.71	48.49	3.44
	2	25.26	7.77	3.55	1.48	11.06	50.89	3.25
	3	22.83	7.62	2.87	1.22	11.02	54.45	3.00
	4	23.23	7.01	2.37	1.12	12.15	54.12	3.31
400W10M-IN	1	24.00	7.95	3.13	1.18	9.61	54.13	3.02
	2	24.68	8.28	2.47	1.11	9.62	53.84	2.98
	3	24.67	8.44	3.30	1.50	9.39	52.70	2.92
	4	24.18	8.29	3.06	1.15	9.38	53.93	2.92

## Conclusions

In this study, we additionally utilized the household microwave oven after the conventional dry-oven curing process, in order to synthesize high-strength CBA-based geopolymers in a time- and cost-efficient manner. The precast samples were prepared by press-molding the raw mixture of the crushed CBA and well-regulated amount of 14 M NaOH activator. These samples were heated in a dry oven at 75 °C for 24 h, after which they were exposed to the microwave energy at powers of 200, 400, 600, 800, or 1,000 W within the adequate time ranges. Not only did we observe the changes in the appearance of samples, but also, we measured the moisture content and body temperature after microwaving. Furthermore, various analytic techniques including SEM-EDS, ATR-FTIR, and RIR-XRD were performed for the morphological, chemical, and crystallographic characterization of the source material and geopolymer products. Therefore, we elucidated the mechanism of the variation in the compressive strength of the CBA-based geopolymers along with the microwave heating by consolidating all the results. The conclusions are as follows:

1. The CBA-based geopolymers achieved a high compressive strength beyond 65 MPa just after a single day of the dry-oven curing, followed by a short time of the microwave heating. The proposed method was proved to be very efficient given the samples achieved the much higher compressive strength than that of the control samples, which were cured at ambient or elevated temperature (75 °C) for 7 and 21 days.
2. As the moisture content of the geopolymers decreased from 14.8% to a critical value (4–6%), as a result of the microwave heating, the compressive strength increased rapidly from 21.0 to above 65 MPa. At this strength-gaining period, the microwave energy evaporated the redundant free water, and it initiated the additional geopolymerization reaction to enhance the macro/microscopic homogeneity.
3. As the moisture content decreased below the critical value (4–6%) via subjection to further microwaving, the compressive strength began to deteriorate. At this strength-losing period, the microwave over-irradiation caused the loss of the chemically bonded water, which led to the high internal stress. Furthermore, thermal runaway occurred as the temperature skyrocketed at the core, and thus, the structural integrity worsened.
4. The microwave energy, therefore, should be employed as far as it does not lead to the evaporation of the chemically bonded water, to achieve the high compressive strength. The optimal point was characterized by a moisture content of 4–6% or an average surface temperature within 120–130 °C.

## Data Availability

The datasets generated during and/or analyzed during the current study are available from the corresponding author on reasonable request.
